# Causes, survival rates, and short-term outcomes of preterm births in a tertiary hospital in a low resource setting: An observational cohort study

**DOI:** 10.3389/fgwh.2022.989020

**Published:** 2023-02-02

**Authors:** Martina Mocking, Kwame Adu-Bonsaffoh, Kwabena A. Osman, Evelyn Tamma, Alexa M. Ruiz, Ruth van Asperen, Samuel A. Oppong, Mirjam Y. Kleinhout, Cynthia Gyamfi-Bannerman, Joyce L. Browne

**Affiliations:** ^1^ Julius Global Health, Julius Center for Health Sciences and Primary Care, University Medical Center Utrecht, Utrecht, Netherlands; ^2^ Department of Obstetrics and Gynaecology, University of Ghana Medical School, Accra, Ghana; ^3^ Department of Child Health, University of Ghana Medical School, Accra, Ghana; ^4^ Department Birth Care, Sint Antonius Hospital, Utrecht, Netherlands; ^5^ Department of Obstetrics, Gynecology, and Reproductive Sciences, UC San Diego, San Diego CA, United States

**Keywords:** prematurity, LMIC, observational cohort, causes, survival, outcomes, longitudinal

## Abstract

**Background:**

Prematurity is the most important cause of death among children under the age of five years. Globally, most preterm births occur in Sub-Saharan Africa. Subsequent prematurity leads to significant neonatal morbidity, mortality and long-term disabilities. This study aimed to determine the causes, survival rates and outcomes of preterm births up to six weeks of corrected age in Ghana.

**Materials and methods:**

An observational prospective cohort study of infants born preterm was conducted in a tertiary hospital in Accra, Ghana from August 2019 to March 2020. Inclusion was performed within 48 h after birth of surviving infants; multiple pregnancies and stillbirths were excluded. Causes of preterm birth were categorized as spontaneous (including preterm pre-labour rupture of membranes) or provider-initiated (medically indicated birth based on maternal or fetal indications). Survival rates and adverse outcomes were assessed at six weeks of corrected age. Recruitment and follow-up were suspended due to the COVID-19 outbreak. Descriptive statistics and differences between determinants were calculated using Chi-squared tests or Kruskal-Wallis test.

**Results:**

Of the 758 preterm deliveries, 654 (86.3%) infants were born alive. 179 were enrolled in the cohort and were analyzed. Nine (5%) were extremely preterm [gestational age (GA) < 28 weeks], 40 (22%) very preterm (GA 28–31 weeks), and 130 (73%) moderate to late preterm (GA 32–37 weeks) births. Most deliveries (*n* = 116, 65%) were provider-initiated, often due to hypertensive disorders in pregnancy (*n* = 79, 44.1%). Sixty-two infants were followed-up out of which fifty-two survived, presenting a survival rate of 84% (*n* = 52/62) at six weeks corrected age in this group. Most infants (90%, *n* = 47/52) experienced complications, predominantly consisted of NICU admission (92%) and interval illnesses (21%) including jaundice and sepsis.

**Conclusions:**

The incidence of adverse outcomes associated with preterm birth in a tertiary facility with NICU capacity is high. Larger longitudinal studies are needed for an in-depth understanding of the causes and longer-term outcomes of preterm birth, and to identify effective strategies to improve outcomes in resource constrained settings.

## Introduction

Preterm birth is the leading cause of neonatal and perinatal morbidity and mortality globally (1–3). Every year, about 15 million babies are born prematurely worldwide, and these rates are rising (1, 4–6). Preterm birth is considered the most important determinant of infant survival and quality of life (2). The highest burden occurs in low-income countries, with an estimated preterm birth rate of 12% in these areas compared to 9.4% in middle- and high-income countries (7–9). Eight out of ten preterm births occur in Sub-Saharan Africa and Southern Asia (4).

Preterm birth complications are the most common cause of death in children under five, leading to approximately a million deaths per year (2, 10). These numbers reflect the need to strengthen the implementation of evidence-based interventions in maternal and newborn healthcare (8, 11). The burden of preterm birth is particularly high during the first 28 days of life, and accounts for 35% of all neonatal deaths and 18% of deaths in children under five years (1, 4, 5, 9). A dramatic survival gap exists between preterm infants born in high- and low-income settings(8), with 50% of infants born at 24 weeks and 90% of infants born at 28 weeks in high-income countries surviving the neonatal period. In contrast, in low-income settings only 10% of those born at 28 weeks of gestation will survive, rising to 50% for infants born at 32 weeks (6, 8).

Among surviving preterm infants, the incidence of neonatal mortality and morbidity is higher compared to those born at term (8). Although morbidity and mortality decrease with each additional gestational week (12), even infants born at 34 to 36 weeks are at increased risk. Short-term complications include respiratory distress syndrome, hypothermia, hypoglycemia, jaundice, infections, necrotizing enterocolitis and intracranial hemorrhage (8, 13–15). Longer term consequences include cerebral palsy, visual and auditory deficits, poor(er) respiratory outcomes, impaired motor and cognitive ability, and symptoms and disorders associated with inattention, anxiety and antisocial behavior (14–18). Beyond their impact on the health and wellbeing of children, prematurity is associated with an economic burden for families, caregivers, health services and other sectors of the economy, due to the increased costs of health care and impact on household's economic activities (16). As such, prematurity remains a major public health concern (7, 16), and it is vital to develop and implement cost-effective interventions and guidelines that ameliorate outcomes of preterm birth (1, 2, 11).

Ghana, a West-African middle-income country, has a prematurity rate of 14.5% (19) and 128,000 infants are born preterm annually. Of these, 8,400 children die due to complications (20). In Ghana, under five mortality was 52 per 1.000 live births in 2017, despite availability of neonatal intensive care (NICU) facilities within the health care system (21, 22). In the country's largest tertiary hospital, rising preterm birth rates are reported up to 18.9% (21). Delineated causes of prematurity are up to 40% provider-initiated (iatrogenic), and often associated with hypertensive disorders in pregnancy (21, 23, 24).

Given the need for LMIC-based studies to better understand the causes, survival, and outcomes of preterm births (25), this study aimed to study these indices within the context of a referral hospital in Ghana.

## Materials and methods

This study was nested in the PETITE study (PrEmaTurIty in Ghana: determinants, clinical course and outcomes of preTErm births in a tertiary hospital in Accra), an observational prospective cohort study. Recruitment was started in August 2019 and follow-up of these infants commenced in November 2019. However, due to the COVID-19 pandemic, research activities were terminated in March 2020 (26). Results are reported in accordance to STROBE recommendations (27).

### Setting

The PETITE study was conducted at Korle-Bu Teaching Hospital (KBTH) at the Departments of Obstetrics and Gynaecology, and Child Health in Ghana's capital, Accra. KBTH is a teaching hospital for the University of Ghana Medical School. The department of Obstetrics and Gynaecology is the largest department and approximately 10,000 deliveries take place annually and accommodates a neonatal intensive care unit (NICU) (21). The department is subdivided into five units that are each led by senior consultants who supervise the equally distributed medical staff, such as residents, house officers and midwives. The NICU has a capacity of 50 incubators and is directed by pediatricians with extensive experience in neonatal care. From 25 weeks of gestation medical care can be provided, including administration of antibiotics, corticosteroids, phototherapy, nasogastric tube feeding and respiratory support by nasal cannula, face mask and continuous positive airway pressure therapy. During this study, surfactant administration, cranial ultrasonography and total parental nutrition were not carried out at the NICU.

### Participants

Eligibility criteria were livebirth preterm infants through any mode of delivery. Other eligibility criteria were maternal age of 18 years or older and provision of written informed consent. Exclusion criteria were multiple pregnancy, stillbirth, and early neonatal death prior to recruitment.

### Study procedures

On a daily basis, research assistants assessed women admitted to the maternity wards that were eligible for inclusion and willing to provide informed consent. Once informed consent was given, information about the woman's socio-economic, last pregnancy and delivery, and (obstetric) history, were obtained from the patient files and by questionnaire. Phone numbers were collected in order to contact participants for follow-up visits located at the Child Health department of KBTH, when their infants reached 6 weeks corrected age. During follow-up, trained research assistants and pediatricians obtained infant medical history and anthropometric information from infants' medical files and by examination. Loss to follow-up was considered when phone numbers were out of order or when participants remained unreachable after several attempts.

**Figure 1:**
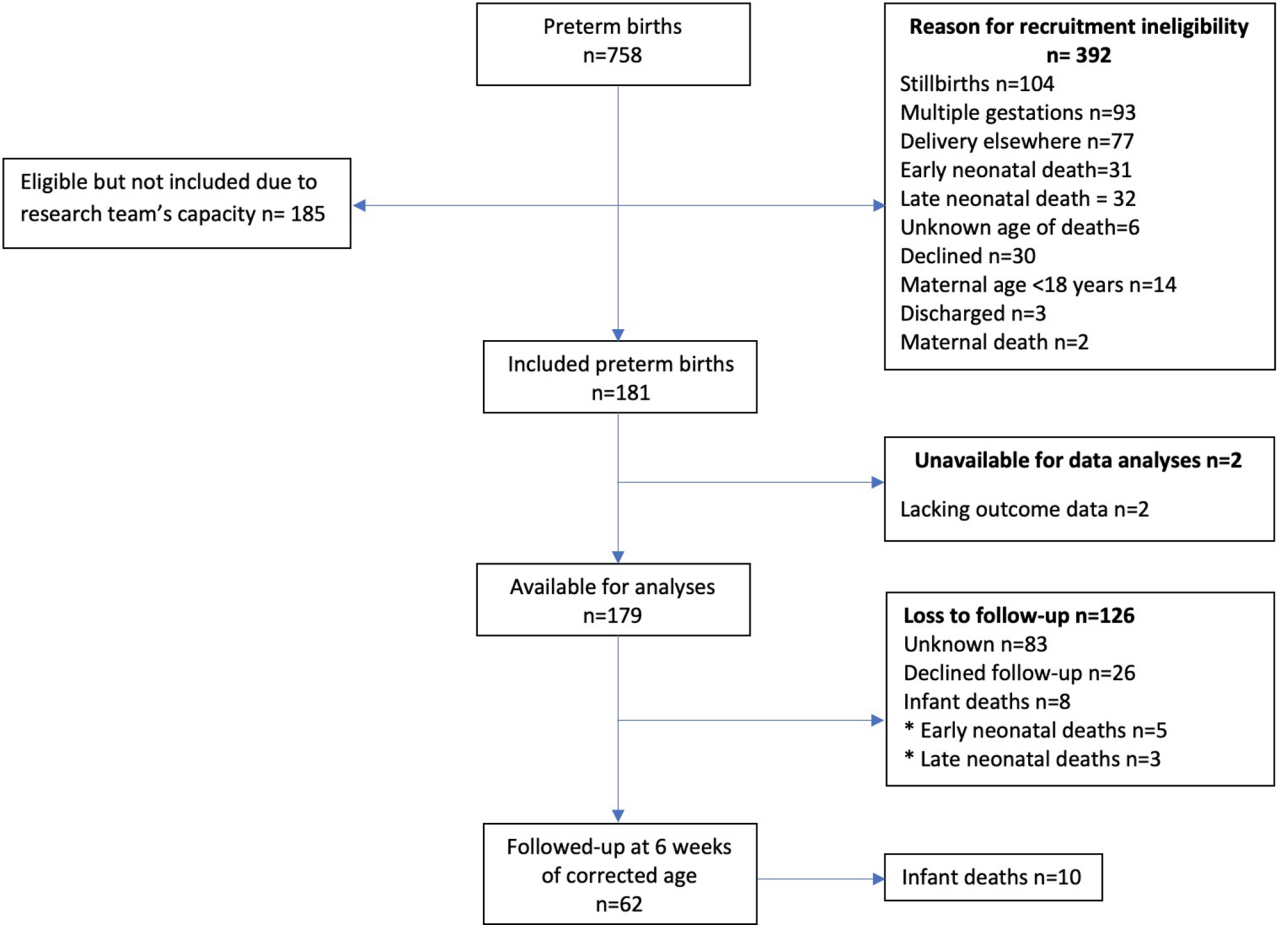
Flowchart indicating recruitment of the study participants.

The outbreak of the COVID-19 pandemic impacted data-collection heavily, as the recruitment of participants and follow-up visits in the hospital were halted. Due to the country's lock-down that followed shortly after (26), accompanied by COVID related restrictions such as restraints in relation to public transport, hampering follow-up visits for participants, PETITE was eventually compelled to stop its study activities. Figure 1 indicates the recruitment process for the study participants.

### Outcomes

The causes of preterm birth were categorized into spontaneous onset of labour (including preterm prelabour rupture of membranes), or provider-initiated birth (medically indicated based on maternal or fetal indications) and measured as an outcome in this study (28–30).

Mortality rates were calculated among excluded and included neonates. Stillbirths were defined as death after 28 weeks of pregnancy occurring before or during birth. Early and late neonatal deaths were defined as infant death occurring within the first week of life and after the first week but before the end of the fourth week of life respectively (31). Neonatal death comprises both early and late neonatal deaths. Perinatal death was defined as the sum of stillbirths and early neonatal deaths (31). The survival rate was calculated up to six weeks of corrected age. Corrected age refers to the postnatal age corrected for the number of weeks that birth occurred before reaching term gestation of 40 weeks, for example an infant born at 36 weeks who is now six weeks old has a corrected age of two weeks (32). Mortality and survival rates were assessed at different stages of the study procedures; before enrolment in the cohort (stillbirth rates, early and late neonatal deaths and unknown age at death), or after enrolment in the cohort (early and late neonatal deaths, survival rates). Data during NICU admission was not included.

To analyze infant morbidity at follow-up, a composite variable for complications was created, consisting of diagnosis of heart murmur, wheezing, and decreased muscle tone, as well as NICU admission, postpartum and/or (emergency) hospital re-admission and interval-illness after discharge. Interval-illness was any form of illness reported by the mother, including (emergency) hospital admission. Additionally, head circumference (cm, centimeters), body length (cm), weight (gr, grams) and umbilical herniation were assessed at follow-up.

### Determinants

Based on the WHO's definition, preterm birth was defined as birth <37 weeks of gestation and categorized as extremely preterm (birth <28 weeks), very preterm (birth between 28 and 31 weeks), or moderate to late preterm (between 32 and 37 weeks of gestation) (6, 30). Generally, gestational age was determined by ultrasound scan at the first antenatal visit (booking) or based on the last menstrual period and confirmed with ultrasound scan.

Maternal determinants included age (years), ethnicity, educational level, current occupation, marital status, current body mass index (BMI), and maternal intoxication during current pregnancy or gave up before or during current pregnancy (including alcohol, nicotine, cigarette, or cigar consumption use as well as exposure to household smoking). Medical and obstetric history were reported, including maternal chronic disease (consisted of before or during pregnancy diagnosed sickle cell disease, diabetes mellitus, respiratory disease, active tuberculosis, epilepsy, renal, hematologic, collagen, adrenal, thyroid or other endocrine disease, hepatitis B infection, human immunodeficiency virus (HIV) or malaria), parity, number of antenatal care (ANC) visits, at least eight or more ANC contacts as recommended by recent WHO guidelines (33), (pre-existent) hypertensive disorders of pregnancy (HDP), antenatal corticosteroid therapy (in the form of dexamethasone either intramuscular or intravenous) and mode of delivery.

For post-partum outcomes, the following infant variables were collected: sex, gestational age at birth, birthweight (g), low birth weight (LBW, <2500 g), body length (cm), small for gestational age (SGA, <10th percentile), Apgar scores at one and five minutes (dichotomized into <7 and ≥7), NICU admission, length of NICU admission (days), need of resuscitation (including nasal cannula, face mask or continuous positive airway pressure therapy), and infant mortality defined as death after hospital discharge reported during follow-up.

### Study size

Based on previously reported data, per month a minimum of 150 eligible preterm births were expected (21). In combination with a predefined recruitment period of three months, a specified sample size of 350 subjects was estimated to obtain adequate power (21, 34) to assess incidences of the composite of complications. However, in March 2020, face-to-face data collection was suspended due to the COVID-19 pandemic (26). Therefore, the current analysis used available data until then, resulting in 181 enrolled participants.

### Data analysis

Women's baseline characteristics and infants' perinatal and short-term outcomes were presented by classification of preterm birth according to the WHO(30). Descriptive statistics with frequencies and percentages for categorical variables or with means (or medians if appropriate due to skewed data) and standard deviations (SD) for continuous variables were used. Differences between determinants were statistically tested using Chi-squared test. Kruskal-Wallis tests, Mann-Whitney U tests and Fisher's exact tests were applied to compare non-normally distributed measurements. Survival rates were calculated and presented with frequencies and percentages.

Maternal, obstetric, and infant (outcome) characteristics of followed-up infants and deceased infants were compared to infants that were lost to follow-up (due to refusal of further participation, infant demise, or for unknown reason) with descriptive statistics. Missing data was not imputed due to low occurrence, and analyses were restricted to women of whom gestational age at birth was reported.

A *p*-value <0.05 was considered as significant. Data were collected by paper questionnaires, entered into REDCap and downloaded into IBM SPSS Statistics (version 21.0 for Windows) to perform statistical analyses.

### Study approval

Approval was obtained from the Ethical and Protocol Review Committee of College of Health Sciences, University of Ghana (CHS-Et/Ml0- 9.2/2018–2019). Written informed consent was obtained from all the mothers prior to enrolment.

## Results

A total of 758 preterm deliveries occurred during the recruitment period. Of these deliveries, 181 (49.5% of the eligible births) were enrolled in the cohort study, and data of 179 (98.9%) infants were available at time of data analysis. This included 8 (4.5%) infant deaths. A total of 26 (14.5%) mothers declined further participation during follow-up (Figure 1). In total, 62 (34.6%) infants were followed up, of which 10 (16.1%) died, and 52 (83.9%) attended follow-up at six weeks of corrected age.

### Maternal and obstetric characteristics

Table 1 presents maternal baseline characteristics of infants included in the cohort by classification of preterm birth. Nine infants (5.0%) were extremely premature, 40 (22.3%) were very premature, and 130 (72.6%) were moderate to late preterm born. On average, women were 30.8 (SD ± 5.8) years old, and more than half were of Akan ethnicity. Most of the women were married (*n* = 129, 72.1%), more than half of them had secondary level education (*n* = 101, 59.4%) and nearly three-quarters of women had an informal occupation (*n* = 133, 74.3%). One-third of the women were nullipara (*n* = 61, 34.1%) and in most women (*n* = 161, 89.9%) gestational age was determined using ultrasound scan. On average women received their first ultrasound at 19 weeks of gestation (mean = 19.1, SD ± 9.6) (not shown in Table) and attended 5.4 (SD ± 2.4) ANC visits of which 17% attended ANC eight times or more (*n* = 27, 16.9%). HDP was reported in almost half of the women (*n* = 79, 44.1%). In total over three-fourths received antenatal corticoid therapy (*n* = 138, 77.1%) and among women with a gestational age below 34 weeks (*n* = 80), 90% received corticoid steroids antenatally (*n* = 72, 90.0%; missing *n* = 1) (not shown in table). The vast majority delivered by caesarean section (*n* = 132, 73.7%).

**Table 1:** Baseline characteristics of the study population.

		Gestational age at birth (weeks)				
Maternal characteristics	Overall *n *= *179*	<28 *n = 9* *n* (%)	28-31 *n = 40* *n* (%)	32-37 *n = 130* *n* (%)	Missing *n* (%)	*P*
Maternal age (years)^a^	30.8 ± 5.8	30.0 ± 4.8	31.0 ± 4.7	30.9 ± 6.2	–	0.86^b^
Maternal age (years)					–	0.91^c^
<35	125 (69.8)	6 (66.7)	29 (72.5)	90 (69.2)		
≥35	54 (30.2)	3 (33.3)	11 (27.5)	40 (30.8)		
Ethnicity					4 (2.2)	
*Akan*	90 (51.4)	4 (44.4)	18 (45.0)	68 (54.0)		
*Ewe*	18 (10.3)	1 (11.1)	2 (5.0)	15 (11.9)		
*Ga*	39 (22.3)	1 (11.1)	16 (40.0)	22 (17.5)		
*Other*	24 (16.0)	3 (33.3)	4 (10.0)	21 (16.7)		
Education					9 (5.0)	0.50^d^
*None/Primary*	22 (12.9)	–	5 (13.5)	17 (13.7)		
*Secondary*	101 (59.4)	5 (55.6)	25 (67.6)	71 (57.3)		
*Tertiary*	47 (27.6)	4 (44.4)	7 (18.9)	36 (29.0)		
Occupation					–	0.48^c^
*Formal*	46 (25.7)	1 (11.1)	9 (22.5)	36 (27.7)	–	
*Informal*	133 (74.3)	8 (88.9)	31 (77.5)	94 (72.3)		
Marital status					–	0.50^c^
*Single*	50 (27.9)	1 (11.1)	11 (27.5)	38 (29.2)		
*Married*	129 (72.1)	8 (88.9)	29 (72.5)	92 (70.8)		
Mean BMI (kg/m^2^)^a^	30.3 ± 6.1	39.7 ± 3.3	29.3 ± 6.0	30.2 ± 6.0	81 (45.3)	0.039^b^
Substance use^e^ (yes)	14 (7.8)	1 (11.1)	2 (5.0)	11 (8.5)	*	0.73^c^
Comorbidities^f^ (yes)	56 (31.3)	2 (22.2)	11 (27.5)	42 (32.3)	–	0.65^d^
*Sickle cell disease*	20 (11.2)	–	1 (2.6)	19 (14.6)	2 (1.1)	
*Malaria*^g^	6 (3.4)	1 (14.3)	1 (3.2)	4 (3.3)	20 (11.2)	
*Human immunodeficiency virus infection*	8 (4.5)	–	2 (5.4)	6 (4.8)	9 (5.0)	
*Hepatitis B infection*	8 (4.5)	1 (12.5)	3 (8.8)	4 (3.2)	11 (6.1)	
*Tuberculosis*^d^	1 (0.6)	–	–	1 (0.77)	–	
Obstetric characteristics						
Parity					1 (0.6)	0.51^c^
*nullipara*	61 (34.1)	4 (44.4)	11 (27.5)	46 (35.7)		
*multipara*	117 (65.4)	5 (55.6)	29 (72.5)	83 (64.3)		
Number of ANC visits	5.4 (2.4)	6.3 (2.7)	4.0 (1.9)	5.7 (2.4)	19 (10.6)	0.001^b^
Gestational age assessed by ultrasound scan (yes)	161 (89.9)	9 (100.0)	33 (86.8)	115 (89.8)	4 (2.5)	0.04^b^
ANC visits						0.077^c^
<8	133 (83.1)	7 (77.8)	30 (96.8)	96 (80.0)	19 (10.6)	
≥8	27 (16.9)	2 (22.2)	1 (3.2)	24 (20.0)		
HDP (yes)	79 (44.1)	5 (44.4)	14 (35.0)	60 (46.2)	**	0.58^d^
Antenatal corticosteroid therapy (yes)	138 (77.1)	9 (100.0)	36 (90.0)	93 (71.5)	1 (0.7)	0.032^d^
Mode of delivery					–	0.49^c^
*Vaginal*	47 (26.3)	3 (33.3)	13 (32.5)	31 (23.8)		
*Cesarean section*	132 (73.7)	6 (66.7)	27 (67.5)	99 (76.2)		

^a^ Presented as mean ± standard deviation (SD).

^b^ Kruskal-Wallis test.

^c^ Chi-squared test.

^d^ Fisher's exact test.

^e^ Describing use of nicotine,cigarettes, cigars, chewing of tobacco or alcohol during current pregnancy or gave up during current pregnancy.

^f^ Including sickle cell disease, diabetes mellitus, respiratory diseases (asthma and cough), tuberculosis, epilepsy, renal or hematologic disease, malaria in pregnancy, HIV, and hepatitis B.

^g^ In pregnancy alone. d. Including active infection.

* Composite variable, including 27.9% missing in exposure to household smoking (*n* = 50, 50/179).

** Composite variable, including 0.6% missing in chronic hypertension (*n* = 1/179).

Mean (current) maternal BMI was 30.3 (SD ± 6.1) and almost a third (*n* = 56, 31.3%) of women had comorbidities, including sickle cell disease (*n* = 20, 11.2%, of which *n* = 19 diagnosed before pregnancy), hepatitis B infection (*n* = 8, 4.5%, of which *n* = 2 diagnosed before pregnancy), human immunodeficiency virus (*n* = 8, 4.5%, of which *n* = 4 diagnosed before pregnancy), malaria in pregnancy alone (*n* = 6, 3.4%), and tuberculosis (*n* = 1, 0.6%). Fourteen (7.8%) women reported substance exposure or use: household smoke exposure (*n* = 9, 64.3%), alcohol use during pregnancy (*n* = 4, 28.6%, of which two women gave up during pregnancy), nicotine use during pregnancy (*n* = 2, 14.3%), and regularly smoking cigarettes before pregnancy (*n* = 1, 7.1%). The mean gestational age at booking = 15.6 weeks ±7.0 SD).

### Survival rates

There were 140 perinatal deaths (18.5%, 185 per 1,000 births) and 654 live births (86.3%, 863 per 1,000 births) among the 758 preterm births that occurred during the recruitment period resulting in a perinatal survival rate (*n* = 618) of 81.5% (815 per 1,000 births). Of these, 104 (13.7%, 137 per 1,000 births) were stillbirths, and 36 (4.7%, 47 per 1,000 births) were early neonatal deaths (of which five occurred after inclusion).

Of the 179 surviving infants included in the preterm birth cohort, eight (4.5%) died before discharge, including five (2.8%) early neonatal and three (1.7%) late neonatal deaths. Of the 62 (34.6%) infants that were followed-up to six weeks corrected age, 52 survived (83.9%). Ten (16.1%) infants died between discharge and follow-up at unknown age, half of these (*n* = 5) were extremely preterm and the other half (*n* = 5) were very preterm born. Mortality rates for moderate to late preterm birth infants were 38 per 1,000 births (3.8%, *n* = 5/130), very premature 150 per 1,000 births (15.0%, *n* = 6/40), and extremely preterm 778 per 1,000 livebirths (77.8%, *n* = 7/9).

### Perinatal outcomes and causes of preterm birth

Table 2 presents perinatal outcomes and the causes of preterm birth. Mean gestational age at birth was 33 weeks (SD ± 2.8) (not shown in Table 2) and most infants were moderate to late preterm (*n* = 130, 72.6%). The proportion of female and male sex were similar (female, *n* = 87, 48.6%). Mean birth weight was 2061.5 g (SD ± 714.4) with an average body length of 44.7 cm (SD ± 5.1). Low birth weight was reported in three-quarters (*n* = 133, 77.8%) of infants and about 10% were small for gestational age (*n* = 19, 11.3%). More than one-third (*n* = 63, 35.2%) of births occurred spontaneously and 64.8% (*n* = 116) were provider-initiated. Low Apgar scores (<7) at one and five minutes were reported in 83 (48.5%) and 34 (20%) of infants. The vast majority (*n* = 159, 89.3%) of the neonates included in the cohort were admitted to the NICU.

**Table 2:** Perinatal outcomes and major causes of preterm birth.

		Gestational age at birth (weeks)				
Perinatal outcomes	Overall *n* = *179* *n* (%)	<28 *n = 9* *n* (%)	28–31 *n = 40* *n* (%)	32–37 *n = 130* *n* (%)	Missing *n* (%)	*P*
Sex of newborn (female)	87 (48.6)	6 (66.7)	19 (47.5)	62 (47.7)	–	0.57^a^
Mean birth weight^b^ (grams)	2061.5 ± 714.4)	1012.9 ± 324.0	1401.7 ± 265.4	2311.5 ± 641.5	8 (4.5)	<0.001^c^
Low birth weight	133 (77.8)	7 (100.0)	37 (100.0)	89 (70.1)	8 (4.5)	<0.001^c^
Mean Length (cm)^b^	44.7 ± 5.1	35.0 ± 2.8	40.2 ± 4.5	45.9 ± 4.5	53 (29.6)	<0.001^c^
Small for gestational age (yes)	19 (11.3)	2 (22.2)	2 (5.4)	15 (12.3)	11 (6.1)	0.25^a^
Cause of preterm birth					–	0.22^d^
*Spontaneous*	63 (35.2)	4 (44.4)	18 (45.0)	41 (31.5)		
*Provider-initiated*	116 (64.8)	5 (55.6)	22 (55.0)	89 (68.5)		
Apgar score <7 at 1 min	83 (48.5)	5 (71.4)	28 (75.7)	50 (39.4)	8 (4.5)	<0.001^a^
Apgar score <7 at 5 min	34 (20.0)	3 (42.9)	16 (44.4)	15 (11.8)	9 (5.0)	<0.001^d^
NICU admission	159 (89.3)	9 (100.0)	40 (100.0)	110 (85.3)	1 (0.6)	0.012^a^
Resuscitation among infants followed up	21 (40.4)	–	6 (15.0)	15 (11.5)	–	0.015^a^
Infant mortality	8 (4.5)				83 (46.4)*	0.011^a^
Early neonatal death	5 (2.8)	2 (28.6)	–	3 (4.3)	–	
Late neonatal death	3 (1.7)	–	1 (5.0)	2 (2.9)	–	

^a^ Fisher's exact test.

^b^ presented as mean ± standard deviation (SD).

^c^ Kruskal-Wallis test.

^d^ Chi-Squared Test.

* Encompassing infants of whose caregivers could not be reached during follow-up.

### Outcomes up to six weeks corrected age

Outcomes of infants up to six weeks of corrected age were assessed for a total of 52 surviving infants (Table 3). On average, these infants were born at 34 weeks' gestation (mean = 33.7 ± 2.1 SD). The majority were born moderate to late preterm (*n* = 42, 80.8%), and nearly 20% (*n* = 10, 19.2%) were very preterm. No extremely preterm born infants attended follow-up; out of the total of nine eligible infants, seven (77.8%) died and two (22.2%) infants were lost to follow-up (Table 4). Women who were followed up delivered more often moderate to late preterm (80.8%, *n* = 42/52), compared to those whose infants died (27.8%, *n* = 5/18) or were lost to follow-up (73.5%, *n* = 61/83).

**Table 3:** Short-term outcomes up to six weeks (corrected age) categorized by classification of preterm birth.

		Gestational age at birth in weeks				
Indicator	Overall *n* = 62 *n* (%)	<28 *n* = 5 *n* (%)	28-31 *n* = 15 *n* (%)	32-37 *n* = 42 *n* (%)	Missing (%)	*P*
Infant mortality	10 (11.4)	5 (71.4)	5 (83.3)	–	–	0.01^a^
Outcomes at follow-up visit	*n* = 52	*n* = 0	*n* = 10	*n* = 42		
NICU admission (yes)	47 (92.2)	–	10 (100.0)	37 (90.2)	1 (1.9)	0.57^a^
Length of NICU admission (days)^b^	12.9 ± 13.9		24.8 ± 20.7	9.8 ± 9.7	–	0.03^c^
Head circumference (cm)^b^	37.8 ± 3.8	–	37.3 ± 2.6	38.0 ± 4.2	1 (1.9)	0.06^c^
Body length (cm)^b^	58.1 ± 4.4	–	55.6 ± 5.2	58.8 ± 4.0	8 (15.4)	0.07^c^
Weight (grams)^b^	5091.7 ± 1333.8	–	4510.0 ± 1310.2	5258.1 ± 1340.7	–	0.13^c^
Heart murmur (yes)	1 (1.9)	–	–	1 (2.4)	–	–
Wheezing (yes)	1 (1.9)	–	–	1 (2.4)	–	–
Umbilical herniation (yes)	15 (28.8)	–	3 (30.0)	12 (28.6)	–	1.0^a^
Decreased muscle tone (yes)	1 (2.0)	–	1 (10.0)	–	2 (3.8)	–
Interval illness (yes)	11 (21.2)	–	5 (50.0)	6 (14.3)	–	0.03^a^
(Emergency) hospital admission (yes)	8 (15.4)	–	3 (30.0)	5 (11.9)	–	0.17^a^
Length of hospital stay (days)^b^	8.4 ± 4.9	–	9.0 ± 1.0	8.0 ± 6.4	–	0.65^c^
Composite of adverse outcomes	47 (90.4)	–	10 (100)	37 (88.1)	–	0.57^a^

^a^ Fisher's exact test.

^b^ presented as mean ± standard deviation (SD).

^c^ Mann–Whitney *U* test.

**Table 4:** Maternal, obstetric, and infant characteristic between followed-up and lost to follow-up infants.

Maternal characteristics	Followed-up *n *= *52* *n* (%)	Declined follow-up *n = 26* *n* (%)	Deceased *n = 18* *n* (%)	Unknown *n = 83* *n* (%)	Missing *n* (%)	*P*
Mean maternal age (years)^a^	31.7 ± 4.7	31.5 ± 6.8	30.4 ± 5.7	30.2 ± 6.1	–	0.55^b^
Maternal age groups (years)					–	0.27^c^
<35	36 (69.2)	15 (57.7)	11 (61.1)	63 (75.9)		
≥35	16 (30.8)	11 (42.3)	7 (38.9)	20 (24.1)		
Ethnicity					4 (2.2)	0.60^d^
*Akan*	24 (48.0)	15 (60.0)	9 (50.0)	42 (51.2)		
*Ewe*	7 (14.0)	1 (4.0)	1 (5.6)	9 (11.0)		
*Ga*	13 (26.0)	3 (12.0)	3 (16.7)	20 (24.4)		
*Other*	6 (12.0)	6 (24.0)	5 (27.8)	11 (13.4)		
Education					9 (5.0)	0.92^c^
*None/Primary*	25 (50.0)	10 (41.7)	11 (61.1)	41 (52.6)		
*Secondary*	12 (24.0)	6 (25.0)	3 (16.7)	15 (19.2)		
*Tertiary*	13 (26.0)	8 (33.3)	4 (22.2)	22 (28.2)		
Occupation					–	0.22^c^
*Formal*	18 (34.6)	8 (30.8)	3 (16.7)	17 (20.5)		
*Informal*	34 (65.4)	18 (69.2)	15 (83.3)	66 (79.5)		
Marital status					–	0.85^c^
*Single*	39 (75.0)	18 (69.2)	14 (77.8)	58 (69.9)		
*Married*	13 (25.0)	8 (30.8)	4 (22.2)	25 (30.1)		
Mean BMI (kg/m^2^)^a^	30.5 ± 6.6	27.8 ± 3.9	31.1 ± 6.3	30.6 ± 6.3	81 (45.3)	0.57^b^
Substance use (yes)^e^	–	2 (7.7)	1 (5.6)	11 (13.3)	–	0.020^d^
Comorbidities^f^ *Yes*	16 (30.8)	8 (30.8)	5 (27.8)	27 (32.5)	–	0.98^c^
Obstetric characteristics						
Number of ANC visits	5.7 (2.2)	4.7 (2.3)	5.4 (2.2)	5.4 (2.6)	19	0.29^a^
ANC visits					19 (10.6)	0.53^d^
<8	38 (84.4)	19 (86.4)	16 (94.1)	60 (78.9)		
≥8	7 (15.6)	3 (13.6)	1 (5.9)	16 (21.1)		
Parity					1 (0.6)	0.96^c^
*nullipara*	18 (34.6)	10 (38.5)	6 (33.3)	27 (32.9)		
*multipara*	34 (65.4)	16 (61.5)	12 (66.7)	55 (67.1)		
HDP (yes)	25 (48.1)	12 (46.2)	10 (55.6)	32 (38.6)	–	0.50^c^
Mode of delivery					–	0.55^c^
*Vaginal*	17 (32.7)	6 (23.1)	4 (22.2)	20 (24.1)		
*Cesarean section*	35 (67.3)	20 (76.9)	14 (77.8)	63 (75.9)		
Perinatal outcomes						
Classification of preterm birth					–	<0.001^d^
*Extremely*	*–*	–	7 (38.9)	2 (2.4)		
*Very*	10 (19.2)	4 (15.4)	6 (33.3)	20 (24.1)		
*Moderate to late*	42 (80.8)	22 (84.6)	5 (27.8)	61 (73.5)		

^a^ presented as mean ± standard deviation (SD).

^b^ Kruskal-Wallis test.

^c^ Chi-square test.

^d^ Fisher's exact test.

^e^ Describing use of nicotine, cigarettes, cigars, chewing of tobacco or alcohol during current pregnancy or gave up during current pregnancy.

^f^ Including sickle cell disease, tuberculosis, malaria in pregnancy, HIV and hepatitis B infection.

Most neonates were admitted to the NICU (*n* = 47, 92.2) with a mean length of stay of approximately 13 days (mean = 12.9, SD ± 13.9) in which very preterm infants were admitted for significantly longer (mean = 24.8, SD ± 20.7) than moderate to late preterm infants (mean = 9.8 ± 9.7 SD) (*p* = 0.03). On average, infants had a head circumference of 37.8 cm (SD ± 3.8), with a body length of 58.1 cm (SD ± 4.4), and mean body weight of 5091.7 g (±1333.8 SD) at six weeks follow up. A heart murmur was diagnosed once (*n* = 1, 1.9%), as well as wheezing (*n* = 1, 1.9%) and decreased muscle tone (*n* = 1, 1.9%). Almost one-third of infants were diagnosed with umbilical herniation (*n* = 15, 28.8%). In 21.2% (*n* = 11) of infants, post-discharge interval-illness was reported which led to re-admission in eight (72.7%) due to jaundice (*n* = 4, 50%), sepsis (*n* = 3, 37.5%) and fever with upper respiratory tract infection (*n* = 1, 12.5%). On average, infants were re-admitted for eight days (mean = 8.4, SD ± 4.9). In most of the infants (*n* = 47, 90.4%) the composite of adverse outcomes was reported.

## Discussion

In this observational cohort study of preterm infants in Ghana, most were born due to provider initiated preterm birth, and nearly all required NICU admission. Live birth rate was >85% whiles perinatal mortality was 18.5%. The survival rates among infants who survived the first 48 h and were followed-up to six weeks of corrected age was nearly 85%, but most of these infants (90%) experienced adverse outcomes, predominately NICU admission and interval-illness.

The perinatal survival rates reported among preterm born infants in this urban middle-income country referral setting seem better compared to some other LMIC settings mentioned in the WHO report “Born too soon”, which shows survival rates at merely 30% in infants born at 28 to 32 weeks and almost no survival in infants born at <28 weeks (8). However, comparing our findings with current literature is challenging, not only because survival rates in “Born too soon” are assessed according to gestational age at birth, also perinatal mortality rates in low resource settings show great variation based on setting, follow-up periods and outcomes definitions (35). To illustrate, reported perinatal mortality rates ranged from 52 per 1,000 births in a district hospital in Tanzania to 460 per 1,000 births in Nigerian infants with mean gestational age of 32 weeks at birth admitted to NICU (35, 36). One recent study, comparable to ours, conducted in a different geographic region of Ghana found a lower survival rate of almost 70% in live preterm born infants admitted to a simplified NICU called “Special Care Baby Unit” (37). This unit lacked several NICU logistics such as machinal ventilation, which may explain the difference in survival rate. The high numbers of provider-initiated preterm births and cesarean sections in our study corresponds with the referral function of the hospital, and its high-risk case load, similar to other tertiary LMICs settings (8, 21, 23, 38, 39). As preterm birth is the most evident risk factor for low Apgar scores, the high number of low Apgar scores that we described correlates with our study population (40–42). In LMICs, the reported incidence of low Apgar scores at five minutes in preterm born infants varies approximately between 10% to 20%, which is in line with our findings (43–46). In high income countries these numbers are evidently better (47, 48).

To assess adverse infant outcomes in this study, physical abnormalities, interval illness and health-seeking behavior were used as proxies in a combined variable for infant morbidity. Apart from restricted growth, no other consensus criteria for morbidity are available for preterm infants (35). Our cohort showed high rates of umbilical herniation occurring in almost one-third of the infants, and this has been observed by others (49, 50). The low numbers for other physical abnormalities could be reflective of the small sample size of followed-up infants, as well as NICU data that was not collected, for example, infant respiratory distress syndrome or necrotizing enterocolitis. Like others, we also observed high interval illness (>20%), of which most infants were re-hospitalized (12, 14, 51). This is not only relevant in the counseling of parents about what to expect in terms of health outcomes when faced with a preterm birth, but also of relevance given the associated long-term financial impact as prolonged and possibly frequent hospitalization is necessary and not always covered through health insurance (8, 52). Whether timing of and conditions at discharge affects these rates we cannot assess based on the available data but could be inquired in future studies.

Evidence-based interventions for preterm and low birth weight infants were recently summarized in a meta-analysis study, including interventions that can be provided prior to referral and in facilities without a NICU (11). Importantly, given the inverse correlation between gestational age and mortality rates (i.e., higher mortality at lower gestational ages) reported within this study and elsewhere (21, 38), and with extremely and very preterm born infants accounting for the majority of deaths (8, 37), interventions targeted at newborns need to be coupled with antenatal care interventions as primary prevention of preterm delivery and to reduce the adverse neonatal outcomes.

### Strengths and limitations

Our study has several strengths. These include the prospective observational design in which infants were followed-up to determine the survival rates of prematurely born infants. In addition, the gestational age was established by ultrasound early in pregnancy or at first antenatal care visit. Whereas ultrasound is considered as the golden standard for gestational age assessment, this method is less common in LMIC studies (30). While most women in Ghana attend antenatal care at a gestational age where reliable gestational age determination is possible by ultrasound, some women do present after their second trimester. This may explain why the NICU discharge for some (very) prematurely born infants in our cohort seems early (e.g., < 34 weeks), because they might have been more matured by assessment. The application of WHO‘s preterm classification systems allows for comparison with international data (8, 30). Most importantly, given the dearth of data from LMIC on the determinants of preterm birth and health consequences (8, 30), our study contributes to bridging this research gap.

Yet, several limitations exist, and these relate particularly to the small sample size, low number of followed-up infants and lacking data of NICU admission. This was a direct result from the abrupt ending of the PETITE study in March 2020 due to the SARS-CoV-2 pandemic, which also withheld us from more extensive research in preterm birth morbidity, as certain adverse outcomes can only be diagnosed after a longer period of time, such as cerebral palsy or developmental disorders. Also, the six weeks follow-up visit was hampered as many mothers were difficult to contact or did not come to follow-up schedules. Therefore, we were forced to assess a composite of adverse infant outcomes at six weeks, which reduces our ability to explore the impact on specific outcomes. Also, it is plausible that the mortality estimates may be an underestimation of the true incidence, due to survivor's bias (women with surviving infants may be more inclined to answer the study team's call or come for a follow up visit), and exclusion of multiple gestations and infants who died <48 h. Also, due to the abrupt ending of follow-up because of study suspension caused by the COVID-19 pandemic, missed cases of post-discharge (early-neonatal) deaths may have occurred, leading to overestimated perinatal survival rates. Furthermore, the generalizability of our findings is limited to higher level facilities, as morbidity and mortality in facilities without extensive NICU and pediatric support can be expected to be higher. Similarly, as followed-up infants were moderate to late preterm born and no extremely preterm infants were represented, the morbidity and mortality estimates for infants born at lower gestational ages can be expected to be higher as well. Lastly, cranial ultrasonography was not performed in this study, as such intraventricular hemorrhage and congenital abnormalities, both resulting in long-term morbidity, could not be diagnosed.

### Research and clinical implications

Globally, one out of every 10 infants is born preterm, of which many will not survive the neonatal period or experience severe morbidities (4, 6, 8, 14, 15). In Ghana, significant challenges exist in the clinical management of preterm birth and prematurity, and partly account for the high disease burden. The clinical challenges may be due to the limited quality research on preterm birth in the country, resulting in lack of locally appropriate evidence to inform clinical policy and improve care (25). In this study, we determined high burden of preterm birth and prematurity related to infant morbidity and mortality. To reduce the high prematurity burden in the country, both observational data as well as intervention studies are needed. However, most studies on prematurity have been conducted in high-income countries and information on outcomes of preterm infants surviving the neonatal period is sparse (4, 8, 25, 53). This study contributes to this data gap and can be the foundation for other studies. Future studies in the interventions to optimize clinical care for women with high risk for preterm birth and care for preterm infants are urgently needed to improve pregnancy outcomes (25). This could include the establishment of specialized clinics for women with high risk of preterm birth to support patient-centered care. Preventive and advocacy measures including client education, women empowerment and effective provider-client communication, could be useful strategies to facilitate early diagnosis and treatment.

### Conclusion

The incidence of adverse outcomes associated with preterm birth in a tertiary facility with NICU capacity is high, despite the availability of specialized neonatal care. Both observational and intervention studies based in LMICs will be necessary to reduce the research gap, gain an in-depth understanding of causes and long-term outcomes of preterm birth, and effectiveness of approaches to improve these in order to achieve Sustainable Development Goal #3—ensuring healthy lives and promoting well-being at all ages.

## Data Availability

The raw data supporting the conclusions of this article will be made available by the authors, without undue reservation.
